# Gender-based differences among ST-elevation myocardial infarction patients in Egypt: secondary analysis from the ACCA-EAPCI ESC-STEMI registry

**DOI:** 10.1186/s43044-025-00671-x

**Published:** 2025-08-04

**Authors:** Sameh Shaheen, Ahmad Wafa, Moustafa Mokarrab, Basem Zarif, Ahmed Bendary, Tarek A. N. Ahmed, Ahmed Rashwan, Mohamed Seleem, Magdy Elmasry, Yasser Abdelhady, Gomaa Abdelrazek, Amr Abdel Aal, Khalid Aly, Mahmoud Saraya, Khaled M. Abd Elaziz, Mahmoud Youssef, Moheb Magdy Wadie

**Affiliations:** 1 Ain Shams University, Cairo, Egypt; 2 Mansoura University, Al-Mansura, Egypt; 3 Al Azhar University, Cairo, Egypt; 4 National Heart Institute, Giza, Egypt; 5 Benha University, Banhā, Egypt; 6 Assiut University, Assiut, Egypt; 7 Fayoum General Hospital, Al-Fayoum, Egypt; 8 Tanta University, Tanta, Egypt; 9 Beni-Suef University, Banī Suwayf, Egypt; 10 Fayoum University, Al-Fayoum, Egypt; 11 Helwan University, Cairo, Egypt; 12 Cairo University, Giza, Egypt

**Keywords:** STEMI, Gender disparities, Egypt, Reperfusion therapy, In-hospital mortality

## Abstract

**Background:**

Gender-based variations in the management and prognosis of ST-segment elevation myocardial infarction (STEMI) have been documented globally, with females often experiencing worse outcomes than males. However, limited data are available on these disparities in Egypt. This research aims to evaluate gender-based variations in the management and short-term findings of STEMI patients in Egypt.

**Results:**

The study analyzed 1,356 STEMI patients, of whom 250 (18.44%) were female. Women were significantly older than men (61.7 ± 11.3 vs. 55.9 ± 11 years; *p* < 0.001) and had a greater prevalence of comorbidities, comprising diabetes (62.8% vs. 35.3%; *p* < 0.001), hypertension (55.2% vs. 33%; *p* < 0.001), and prior stroke/transient ischemic attack (7.2% vs. 3.3%; *p* < 0.01). Women experienced longer delays in seeking care (symptom onset to first medical contact time: 4.2 ± 6.1 vs. 3.1 ± 3.7 h; *p* < 0.001) and tended to show up with advanced heart failure (Killip class III/IV: 12.8 vs. 6.9%; *p* = 0.005). Despite similar rates of primary percutaneous coronary intervention utilization, women had greater rates of in-hospital mortality (7.6 vs. 4.0%; *p* = 0.01). Multivessel disease was less common in women, but they had a greater prevalence of right coronary artery involvement as the culprit lesion (32.9 vs. 21.7%; *p* = 0.002). Medication prescription rates at discharge revealed that women received high-intensity statins (93.2 vs. 96.1%; *p* = 0.04) and ticagrelor (3.6 vs. 7.8%; *p* = 0.01) less commonly.

**Conclusions:**

Egyptian women with STEMI exhibit significant delays in presentation, a greater burden of comorbidities, and worse in-hospital outcomes compared to men.

## Background

Cardiovascular disease (CVD) remains the main reason of morbidity and mortality globally, with women experiencing worse outcomes following acute coronary syndrome (ACS) compared to men [1]. In the Middle East and North Africa (MENA) region, Egypt is responsible for over 15% of cardiovascular-related deaths [2, 3]. Notably, CVD made up 46.2% of total mortality in Egypt in 2017 [4]. However, despite the significant burden of CVD, there are few research in Egypt examining how gender affects ACS clinical presentation and outcomes [5].

Concerning the initial Gulf Registry of Acute Coronary Events (Gulf RACE), there are notable gender variations in the treatment and prognosis of ST-segment elevation myocardial infarction (STEMI) and non-ST-segment elevation myocardial infarction (NSTEMI) [6, 7]. Prior Arabian Gulf registries that included patients with both STEMI and NSTEMI have repeatedly demonstrated that women who present with ACS had greater in-hospital death rates than males [1].

Several prognostic factors, like advanced age at presentation, a greater burden of comorbidities, and the presence of atypical symptoms, have been identified as contributing to delays among STEMI women seeking therapy. These atypical symptoms are associated with delayed diagnosis and may hinder timely management, resulting in poorer clinical outcomes [8]. Investigating possible gender variations in STEMI outcomes in Egypt is crucial, as existing studies are constrained by small sample sizes across various age subgroups of women and men [5].

Therefore, this study aims to evaluate gender-based differences among STEMI patients in Egypt, specifically focusing on potential inequities in management and their influence on short-term outcomes.

## Methods

The European Society of Cardiology ACCA-EAPCI Registry on ST-Elevation Myocardial Infarction (ESC-STEMI Registry) is a collaborative effort by the European Society of Cardiology's European Association of Percutaneous Cardiovascular Interventions (EAPCI) and the Acute Cardiovascular Care Association (ACCA). It is a general, prospective, multicenter, and observational registry designed to determine the therapy and outcomes of STEMI across Europe and Mediterranean countries. The design of ESC-STEMI registry has been detailed in a prior publication [9].

The EURObservational Research Programme (EORP) department at the European Heart House coordinated operational aspects of the project, supported the participating centers and monitored the survey’s methodology. The European Heart House was where the database was safely kept and examined.

With information from 19 Egyptian centers, the Egyptian Society of Cardiology, an ESC member, took part in this registry. The purpose of site selection was to select centers in different geographic locations and with varying degrees of complexity in order to get a sample that is representative of the Egyptian population. 15/17 centers (88.24%) had onsite PCI capabilities, 11/17 centers (64.71%) had onsite cardiac surgery, and 8/16 (50.00%) said they often treated STEMI patients with primary PCI. A median of 91.5 (35.0; 325.0) beds, 500.0 (250.0; 720.0) MI volume, 800.0 (400.0; 2000.0) total PCI, and 220.0 (90.0; 350.0) total main PCI were found in these centers in Q1 and Q3. In randomly chosen centers, local audits were conducted to verify procedure adherence and assess data quality and consecutiveness.

Patients having ST-segment elevations or new onset LBBB in the diagnostic ECG, as well as chest pain or similar symptoms lasting longer than 20 min over the 24 h prior to hospital admission, were the target group. Each center was required to enroll up to 60 consecutive patients. We compared 1356 (15.45%) STEMI patients in the Egyptian cohort with 7420 patients from other European Union nations. The period from March 2016 to February 2018 was when patients were recruited [10].

This research involves a secondary analysis of the Egyptian cohort of the ESC-STEMI registry, specifically comparing gender-based variations in clinical presentation, management, and type of reperfusion therapy, and in-hospital outcomes, of male and female STEMI patients.

## Statistical analysis

Continuous variables were displayed as means with standard deviations or medians with interquartile ranges (IQR) for skewed distributions. Categorical variables were expressed as numbers and percentages and analyzed utilizing the χ^2^ test. Comparisons of continuous variables were conducted using Mann–Whitney U test, while the Kruskal–Wallis test was applied for comparisons involving above two groups. The statistical significance threshold was set at a two-sided *p*-value of < 0.05.

## Results

### Demographic characteristics

Among the 1356 patients, 250 (18.44%) were female, a substantially lower proportion than STEMI cohorts in other countries. Females were significantly older than males (61.7 ± 11.3 vs. 55.9 ± 11 years; *p* < 0.001), with a greater proportion aged over 60 years (59.2 vs. 36.6%; *p* < 0.001) and over 75 years (12.4 vs. 4.6%; *p* < 0.001). Females had significantly higher BMI (31.9 ± 6.5 vs. 28.4 ± 4.2 kg/m^2^; *p* < 0.001), with 57.9% of females having a BMI ≥ 30 kg/m.^2^ compared to 8.3% of males (*p* < 0.001). Comorbidities were more prevalent in females, including previous stroke/TIA (7.2 vs. 3.3%; *p* < 0.01), diabetes (62.8 vs. 35.3%; *p* < 0.001), hypercholesterolemia (16 vs. 9.1%; *p* < 0.001), sleep apnea (2.8 vs. 0.3%; *p* < 0.001), and hypertension (55.2 vs. 33%; *p* < 0.001) Table 1.

**Table 1:** Comparison of demographic, clinical, and treatment outcome findings between female and male STEMI patients

	Females (n = 250, 18.4%)	Males (n = 1106, 81.6%)	*P*-value
Age (years)	61.7 ± 11.3	55.9 ± 11	< 0.001
BMI	31.9 ± 6.5	28.4 ± 4.2	< 0.001
Previous stroke/TIA	7.2%	3.3%	< 0.01
Diabetes	62.8%	35.3%	< 0.001
Hypercholesterolemia	16%	9.1%	< 0.001
Hypertension	55.2%	33%	< 0.001
Smoking	3.6%	71.4%	< 0.001
Atrial fibrillation on qualifying ECG	5.6%	3.0%	= 0.04
Killip class IV on admission	4.8%	2.4%	= 0.004
LDL-cholesterol (mg/dL)	140.3 ± 47.7	132.7 ± 40.6	= 0.05
Mean plasma glucose (mg/dl)	211.5 ± 91.5	174.1 ± 75.7	= 0.001
Time from symptoms onset and FMC (hours)	4.2 ± 6.1	3.1 ± 3.7	< 0.001
Time from FMC to ECG (minutes)	15.3 ± 74.8	16.7 ± 64.7	= 0.8
Time from FMC to primary PCI (hours)	1.7 ± 3.4	2.1 ± 4.2	= 0.3
Time from FMC to thrombolysis (hours)	1.5 ± 1.9	1.9 ± 3.7	= 0.4
No-reperfusion therapy	9.6%	7.4%	= 0.02
Primary PCI	53.6%	48.1%	< 0.02
Thrombolytic therapy	36.8%	44.5%	< 0.02
BMS	28.9%	29.6%	= 0.1
DES	70.4%	70.4%	= 0.1
Serious bleeding	5.6%	5.7%	= 0.6
Blood transfusion	0.8%	0.4%	= 0.3
Cerebrovascular accident	0.0	0.4%	= 0.4
Reinfarction	0.4%	0.9%	= 0.6
Definite stent thrombosis	0.4%	0.0	= 0.06
Mechanical complications	0.8%	0.3%	= 0.2
ECG at discharge showing AF	2.0%	0.9%	= 0.03
Echocardiographic LV EF before discharge	48.9% ± 9.6%	50.7% ± 9.5%	= 0.008
Killip class III in hospital	4.0%	2.7%	= 0.005
Killip class IV in hospital	8.8%	4.2%	= 0.005
In-hospital mortality	7.6%	4.0%	= 0.01
No-reperfusion,	19.0%	16.4%	= 0.7
Primary PCI	5.1%	1.8%	= 0.01
Thrombolysis	4.5%	1.5%	= 0.03
Planned rehabilitation	20.4%	18.6%	= 0.03
Discharge prescription			
Aspirin	92%	95%	= 0.1
Clopidogrel	87.6%	87.2%	= 0.05
Diuretics	17.2%	10.3%	< 0.001
MRA	12.8%	8.9%	= 0.02
PPI	50.8%	46.0%	= 0.03
Ticagrelor	3.6%	7.8%	= 0.01
Statins	93.2%	96.1%	= 0.04
Beta-blockers	78.8%	79.5%	= 0.1
ACE inhibitors	77.2%	82.1%	= 0.09
ARBs	5.6%	2.6%	= 0.007

*BMI*: Body Mass Index; *TIA*: Transient Ischemic Attack; *ECG*: Electrocardiogram; *LDL*: Low-Density Lipoprotein; mg/dL: Milligrams per Deciliter; *FMC*: First Medical Contact; *PCI*: Percutaneous Coronary Intervention; *BMS*: Bare-Metal Stent; *DES*: Drug-Eluting Stent; *AF*: Atrial Fibrillation; *LV EF*: Left Ventricular Ejection Fraction; *MRA*: Mineralocorticoid Receptor Antagonists; *PPI*: Proton Pump Inhibitors; *P*-value < 0.05: Statistically Significant

### Mode and time of presentation to hospital

Ambulance utilization for hospital transport was notably low among STEMI patients. Consequently, the predominant first medical contact was hospital emergency room (ER) staff rather than medical or paramedical ambulance staff, with similar rates between females and males (83.2% vs. 81.0%; *p* = 0.8). Males were more frequently admitted to non-PCI-capable hospitals compared to females (43.4 vs. 36.0%; *p* = 0.03). Females experienced significantly longer delays in seeking medical help after symptom onset compared to males (3.5 ± 6.1 h vs. 2.7 ± 3.7 h; *p* < 0.006). Similarly, the time from symptom onset to the first medical contact (FMC) was greater for females than for males (4.2 ± 6.1 h vs. 3.1 ± 3.7 h; *p* < 0.001) Table 1.

### Management during hospital stay

#### Type of reperfusion

Primary PCI was more frequently offered to females than males (53.6 vs. 48.1%), while thrombolytic therapy was more prevalent among males (44.5 vs. 36.8%; *p* < 0.02). More females than males received no-reperfusion therapy (9.6% vs. 7.4%; *p* = 0.02). Thrombolytic therapy was predominantly administered in the CCU for both genders (98.9% of females vs. 97.0% of males; *p* = 0.5) Table 1.

#### Coronary angiography

No significant variations between females and males were existed regarding prevalence of non-significant lesions upon coronary angiography (4.4 vs. 4.6%; *p* = 0.2) or significant left main disease (1.6 vs. 1.7%; *p* = 0.2). However, a significant variation was noted in the distribution of culprit vessels. Females had a lower prevalence of LAD as the culprit vessel compared to males (54.6 vs. 62.0%) but a greater prevalence of RCA (32.9 vs. 21.7%; *p* = 0.002). Females with STEMI had a significantly higher prevalence of multivessel disease compared to males (51.7 vs. 40.6%; *p* = 0.01) Tables 1 and 2.

**Table 2:** Coronary angiography findings between female and male STEMI patients

Vessel Disease	Females (*n* = 250)	Males (*n* = 1106)	*P*-value
Single-vessel disease	48.3%	59.4%	= 0.01
Multiple vessel disease	51.7%	40.6%	

*P*-value < 0.05: Statistically Significant

#### PCI procedure

Radial access was used in 6.4% of females in contrast to 5.0% of males (*p* = 0.1). The difference in the use of thrombectomy during primary PCI between females and males was insignificant (4.8 vs. 5.1%; *p* = 0.1). Similarly, stent use during PCI did not differ significantly (56.8% in females vs. 50.5% in males; *p* = 0.1). Among patients receiving stents, the type of stent used was comparable, with 28.9% and 70.4% receiving BMS and DES, respectively, among females and 29.6% and 70.4% among males (*p* = 0.1) Table 1.

#### Patient course during hospitalization

The mean left ventricular ejection fraction (LVEF) by echocardiography before hospital discharge was significantly reduced in females in contrast to males (48.9% ± 9.6% vs. 50.7% ± 9.5%; *p* = 0.008). Staged PCI during the index hospitalization was done more often on women than on men (4.0 vs. 1.4%; *p* = 0.04) Table 1.

#### Complications during hospitalization

No significant variations between females and males were existed regarding complications during the hospital course. However, atrial fibrillation detected on discharge ECG was significantly more frequent among females than males (2.0 vs. 0.9%; *p* = 0.03). During hospitalization, females were more likely to present with higher Killip classes. The prevalence of Killip class III and IV was 4.0% and 8.8% in females compared to 2.7% and 4.2% in males, respectively (*p* = 0.005) Table 1.

#### In-hospital mortality and hospital discharge

In-hospital mortality was significantly greater in females than males (7.6 vs. 4.0%; *p* = 0.01) and remained consistently higher across all reperfusion modalities. Mortality was 19.0 vs. 16.4% (*p* = 0.7) among those without reperfusion, 5.1 vs. 1.8% (*p* = 0.01) for those treated with primary PCI, and 4.5 vs. 1.5% (*p* = 0.03) for those receiving thrombolysis Table 1.

The mean hospital stay was similar for females and males (2.5 ± 2.5 vs. 2.65 ± 10.4 days; *p* = 0.8), as was the mean time from admission to death (1.47 ± 3.3 vs. 1.3 ± 2.1 days; *p* = 0.8). Discharge destinations differed significantly, with 88.8% of females discharged home, 2.8% transferred to another hospital, and 7.6% missing information, compared to 92.3%, 3.6%, and 4.0%, respectively, for males (*p* = 0.02). Planned rehabilitation was more typical in females (20.4 vs. 18.6%; *p* = 0.03) Table 1.

Logistic regression analysis identified age > 75 years, diabetes, atrial fibrillation, absence of reperfusion therapy, and Killip class IV as significant independent predictors of in-hospital mortality among STEMI patients Table 3.

**Table 3:** Logistic regression model for prediction of mortality among patients after PCI intervention

Variable	OR (95% CI)	P-value
Gender	0.9 (0.3–2.8)	0.8
Age > 75	7.3 (2.6–20.5)	< 0.001
Smoking	1.0 (0.3–2.6)	0.9
Diabetes	3.9 (1.2–12)	0.02
Hypertension	2.3 (0.7–7.1)	0.1
Metabolic syndrome	0.5 (0.2–1.1)	0.1
AF	4.2 (1.1–15.2)	0.02
No-reperfusion	5.8 (2.1–15.7)	< 0.001
Killip 4 in hospital	176 (75–414)	< 0.001
Delay to FMC above 2 h	1.1 (0.5–2.5)	0.7

Pseudo R^2^ = 0.6; Hosmer and Lemeshow test *P* = 0.84; *P*-value < 0.05 considered statistically significant; *OR*: Odds Ratio; *CI*: Confidence Interval; *AF*: Atrial Fibrillation

#### Medications at hospital discharge

At discharge, no significant variations were existed in the prescription rates of aspirin (92.0% vs. 95.0%; *p* = 0.1) or clopidogrel (87.6% vs. 87.2%; *p* = 0.05). However, females were less likely to be prescribed ticagrelor (3.6% vs. 7.8%; *p* = 0.01) and statins (93.2% vs. 96.1%; *p* = 0.04) but had a higher chance of getting diuretics (17.2% vs. 10.3%; *p* < 0.001), mineralocorticoid receptor antagonists (12.8% vs. 8.9%; *p* = 0.02), and proton pump inhibitors (50.8% vs. 46.0%; *p* = 0.03) Table 1.

## Discussion

In our research, STEMI females were older than males and had more comorbidities, including higher prevelance of atrial fibrillation, lower hemoglobin, higher LDL-cholesterol, and higher plasma glucose levels. These factors, coupled with advanced age, likely contributed to their worse prognosis. Notably, Egyptian females with STEMI were significantly younger than their Western counterparts, being approximately 13 years younger than females in a Swiss study [41], likely because of a greater frequency of risk factors and earlier onset of aggressive atherosclerosis in our population.

Similar to our findings, numerous studies on sex variations in ACS consistently report that women are older and possess a greater burden of comorbidities in contrast to men. A USA study [11] of 78,254 AMI patients (2001–2006) found women were older and had more comorbidities. More recent USA data [12] from 413,500 STEMI hospitalizations confirmed higher comorbidity rates in women, except for smoking and prior sternotomy. Studies from Switzerland [13], Germany [14], and the Netherlands [15] also exhibited that ACS women were significantly older and possessed greater rates of hypertension and diabetes. In Asia, the ACS QUIK Trial [16] in Kerala (India), with data from 21,374, reported women were older (65 vs. 58 years) with a greater incidence of diabetes and hypertension, while Malaysian NCVD-ACS registry [17] found that women were older and were at a greater risk of diabetes, hypertension, dyslipidemia, and renal failure. Similarly, a study from Taiwan [18] on AMI patients undergoing PCI or CABG and the Saudi SPACE registry [19] with 17 participating hospitals both noted women were older and were at greater rates of diabetes, hypertension, and hyperlipidemia.

Risk factors not only affect women more frequently but also have a greater impact on them. For example, diabetes increases MI risk in women with ACS fourfold (OR 4.26) compared to 2.5-fold in men (OR 2.67), with similar patterns observed for hypertension and obesity [12]. Frailty, more common in women, contributes to worse outcomes and lower rates of interventional treatment [20]. The older age of women with ACS can be attributed to estrogen’s protective effect, delaying atherosclerosis until menopause [21]. Post-menopausal estrogen withdrawal worsens cardiometabolic profiles through dyslipidemia, hypertension, insulin resistance, and sedentary behavior, increasing CVD risk [22]. The role of hormone replacement therapy in mitigating this risk remains uncertain [23].

In our study, both males and females with STEMI underutilized EMS/ambulance services, with females experiencing significantly lengthier intervals between symptom onset and seeking medical assistance. The underuse of EMS in Egypt likely reflects inefficiencies in the emergency system, including delayed or absent responses, leading many patients to prefer self-transport to hospitals. Cultural factors, such as embarrassment at being seen in an ambulance, especially in crowded communities, may also contribute. Globally, similar trends have been reported. Mosca et al. [24] noted that only 53% of females with ACS symptoms call an ambulance, and a meta-analysis [25] showed longer median delays in women (1.8–7.2 h) compared to men (1.4–3.5 h). Moreover, the Kerala (ACS QUIK) trial [16] showed that females, compared to males, had significantly longer symptom-to-door times (300 versus 238 min; *P* < 0.001).

Women with ACS often misinterpret symptoms, attributing them to non-cardiac causes, and are more prone to exhibit atypical symptoms like upper back, arm, or jaw pain, dyspnea, or nausea [26, 27]. Studies such as GENESIS-PRAXY [28] and National Registry of Myocardial Infarction [29] found women appeared more frequently without chest pain, especially younger women. In contrast, VIRGO [30] discovered that while the percentage of men and women experiencing angina-like chest discomfort was comparable (83 vs. 77%), women were more likely to exhibit nausea (45%) and dyspnea (44%).

In our study, no significant variation between females and males in the prevalence of non-significant lesions (MINOCA) or significant left main disease, likely due to the small sample size limiting the ability to detect such differences. However, sex differences in ACS pathophysiology are well-documented. A study showed women disproportionately affected by MINOCA, constituting 50% of these cases compared to 25% in AMI with obstructive CAD [31]. Microvascular heart disease is more typical in older female, while younger women, particularly during the reproductive phase, have a fivefold greater incidence of MINOCA than men (14.9 vs. 3.5%) [32]. Women also exhibit structural differences, including smaller coronary arteries and the presence of diffuse non-obstructive plaques. A large Portuguese ACS registry [33] demonstrated that women had a greater probability of normal angiograms (11.7 vs. 5.7%; *p* < 0.001) and single-vessel disease, while men were more often diagnosed with multivessel or significant left main disease (7.1 vs. 6.2%; *p* = 0.002). Additionally, spontaneous coronary artery dissection (SCAD) is a leading reason for AMI in young women (≤ 50 years), accounting for up to 35% of cases and 43% of pregnancy-associated MI [34]. Takotsubo syndrome predominantly affects post-menopausal women, further illustrating unique etiologies of ACS in females [35].

In our study, non-reperfused patients were significantly more common among females, likely due to delayed presentation beyond the reperfusion window, though a gender bias toward less invasive approaches cannot be excluded. Non-reperfused patients also possessed the greatest rate of in-hospital death. However, no significant variations were noted in ECG timing, FMC-to-PCI, or FMC-to-thrombolysis intervals, indicating no logistical delays for females. Among reperfused patients, females and males showed no significant differences in primary PCI details. Females were significantly more likely to undergo staged PCI during the index hospitalization and slightly more likely to undergo CABG, though the latter was not statistically significant. Delays from FMC to reperfusion were similar for both pPCI and thrombolysis. This suggests a need for improvement, as thrombolytics are often given only in the ICU rather than promptly in the ambulance or ER, contributing to unnecessary delays.

Contemporary studies highlight persistent gender disparities in STEMI management. A German study [36] pointed out that women had a lower chance of getting PCI and DES than men, findings echoed by analyses from Switzerland [13] and Latin America [37], where women with STEMI were provided thrombolysis, primary PCI, and CABG at reduced rates. Similarly, US data [38] showed sex-based disparities in the diagnostic coronary angiography procedure, PCI, and CABG, with female patients also experiencing significant delays in door-to-balloon and FMC-to-balloon times [39]. Despite higher bleeding risks in women, the trans-radial approach remains underutilized, contributing to their increased bleeding complications [40]. Women also have a lower likelihood of being given glycoprotein IIb/IIIa inhibitors or offered mechanical circulatory support devices, like intra-aortic balloon pumps, Impella®, or ECMO [36].

The underutilization of radial access observed in this registry for both male and female patients may be attributed to insufficient training opportunities for cardiologists and the inherent learning curve associated with mastering the radial approach. Additionally, in many centers, the radial approach incurs slightly higher costs, and the availability of specialized radial sheaths and catheters is not consistently ensured. However, recent data indicate a growing adoption of radial access in line with guideline recommendations.

A US study of 413,500 STEMI hospitalizations [12] found women did not have as many angiography procedures (81.0% vs. 87.0%) or pPCI (74.0% vs. 82.0%). However, pPCI reduced mortality similarly in women (9.5%) and men (8.4%) across all age groups, with no difference in its effect on duration of hospitalization. Additionally, females have a reduced chance of getting pPCI for STEMI in the ACS QUIK Trial from Kerala, India, with data from 21,374 patients [16]. A Malaysian study [17] found females have a reduced chance of getting pPCI or fibrinolysis with longer door-to-needle and door-to-balloon times. Similarly, data from a Swiss registry [41] reported females were at higher risk of pPCI being withheld, especially younger women (adj. HR 9.31 vs. 4.91 for younger men). Women in Switzerland also underwent fewer techniques for diagnosis and treatment, such as coronary angiography, PCI, and CABG [13].

A German study [14] revealed men received PCI earlier across all age groups, while women aged ≥ 75 years lagged three years behind men in receiving antiplatelet agents like ticagrelor or prasugrel after guideline recommendations. In another study [42], females had a reduced likelihood of revascularization, with similar disparities observed among Black and Hispanic men. Furthermore, various studies reported that female patients were less likely to receive multiple guidelines-directed medical therapies [43–47].

In our study, DAPT prescription rates for aspirin and clopidogrel were similar between genders, but ticagrelor was less frequently prescribed to females, likely due to concerns about bleeding risk. Females were also more commonly prescribed PPIs. Statins were less frequently prescribed to females despite being a class I recommendation, while higher rates of diuretics and MRA in females likely indicate a greater prevalence of heart failure. Within 90 days and one year following the acute incident, female ACS patients are consistently less likely to get medical therapy guided by guidelines, such as beta-blockers, statins, renin-angiotensin system blockers, and antithrombotic medications [20, 26, 28, 30]. Women experience lower prescription rates of these medications, as shown in studies from Korea [48] and Malaysia [17], and also have a reduced chance of getting high-intensity statins post-ACS [49]. Furthermore, adherence to and tolerance of lipid-lowering therapies are poorer in women, with female sex identified as an independent predictor of statin intolerance in meta-analysis [50]. These disparities persist even among women with ACS exhibiting after out-of-hospital cardiac arrest or with preserved LV EF (> 44%) [51].

Consistently, an Italian cohort of 90,779 ACS patients [52] revealed that women were older and possessed a worse medical profile at presentation, potentially explaining their higher in-hospital mortality. Women were less frequently prescribed DAPT (60% vs. 74% in men) and showed lower adherence to drug therapies, consistent with prior studies [53, 54]. Women also utilized cardiac rehabilitation programs less than men despite their proven benefits for long-term outcomes [55]. Similarly, data from the Australian CONCORDANCE registry [56] showed that women with STEMI were older (66.6 vs. 60.5 years) and possessed more hypertension, diabetes, and comorbidities like CKD and dementia but had a reduced likelihood of having previously CAD, PCI, or CABG. Women underwent coronary angiography, revascularization, and pPCI at significantly lower rates, with delays in timely interventions. At six months, women had higher rates of MACE (aOR, 2.68) and mortality (aOR, 2.17). Fewer women were prescribed statins, beta-blockers, or cardiac rehabilitation referrals at discharge.

In the present research, in-hospital mortality was significantly greater in females across all reperfusion modalities and among non-reperfused patients. Females possessed a greater prevalence of heart failure, with more Killip class III/IV cases, lower LVEF, and higher rates of AF rhythm on discharge ECG, contributing to their increased mortality. Numerous studies regularly demonstrate that ACS women, particularly STEMI, experience worse in-hospital and long-term findings in contrast to males [57–60]. A meta-analysis revealed higher short-term mortality for STEMI females (RR 1.24, 95% CI 1.11–1.38, *p* < 0.001) [61]. Similarly, the NCDR-ACTION registry [62] and ACS QUIK Trial [16] reported significantly greater rates of in-hospital and 30-day MACE for women (adjusted RR 1.53 and 1.39, respectively).

In our study, it is believed that while LVEF is an important determinant of in-hospital outcomes, it is not the sole factor. Reperfusion status plays a critical role, with our data showing a higher incidence of no-reperfusion among females, largely attributable to delayed presentation. Additionally, a higher prevalence of comorbidities such as diabetes, hypertension, and particularly atrial fibrillation may further worsen outcomes. Nonetheless, we consider that even the small but statistically significant difference observed in LVEF is likely to have a meaningful impact on prognosis.

Likewise, studies from Malaysia [17], Switzerland [13], and Saudi Arabia [19] showed higher mortality rates for women, particularly for STEMI. In Germany [14], female sex was an independent predictor of in-hospital mortality for patients aged ≥ 75 years, whereas younger females with NSTEMI had greater unadjusted mortality in contrast to males. Long-term outcomes also favor men. EPICOR and EPICOR Asia registries [63] noted higher mortality in women during follow-up, though adjustments for income inequality and country wealth modified these results. VIRGO trial data [32] revealed women had worse quality of life and higher recurrent angina rates 12 months post-ACS. Socioeconomic factors, poorer adherence to guideline-directed therapy, and distinct cardiac pathobiology likely contribute to these disparities. Younger women (< 60 years) consistently exhibit worse outcome in contrast to males of the same age [64].

Women with ACS that had poorer short-term findings may result from advanced age, frailty, and a greater comorbidities burden. Atypical presentations and delayed recognition prolong pre-hospital and reperfusion times, increasing mortality [28, 65–67]. In a US registry, females possessed greater in-hospital mortality for STEMI (7.4% vs. 4.6%) and NSTEMI (4.8% vs. 3.9%, *P* < 0.001), with greater delays and reduced rates of guideline-directed therapy [68]. Younger women (< 65 years) are particularly affected by delayed care and worse mental health recovery post-MI, impacting their quality of life [27, 58, 69].

In line with our results, a Netherlands study [15] showed females were older (68.5 vs. 63.0 years, *p* < 0.001) with more hypertension diabetes and possessed higher one-year mortality (7.3% vs. 5.6%, *p* < 0.001), especially those ≤ 71 years, while older females possessed greater mortality than men (*p* < 0.001). Endothelial dysfunction and plaque erosion are caused by cardiovascular risk factors that are more prevalent in younger women, comprising as obesity and smoking [70, 71]. They are also more likely to present atypically, delaying diagnosis and reperfusion. Extended exposure to coronary heart disease and reduced life expectancy may contribute to excess mortality in older men. A US study of 78,254 AMI patients (2001–2006) [11] found greater unadjusted in-hospital mortality in women (8.2% vs. 5.7%, *P* < 0.0001), largely due to excess deaths in the first 24 h, with adjusted differences persisting in STEMI (10.2% vs. 5.5%, OR 1.12, 95% CI 1.02–1.23). Females were less probable to receive timely aspirin, beta-blockers, reperfusion, or revascularization.

This study is limited by its reliance on secondary analysis of registry data, which may introduce selection bias and unmeasured confounders. The observational design precludes establishing causal relationships, and the data may lack granularity on certain patient-specific factors, such as socioeconomic status or adherence to prescribed therapies. Additionally, the relatively small sample size of female patients might limit the generalizability of the findings and reduce the statistical power to detect some gender-specific differences. Future studies should include larger, prospectively collected cohorts with a focus on socioeconomic and psychosocial variables to better elucidate the observed disparities. Steps to improve the outcomes of women presenting with chest pain and acute coronary syndrome in Egypt should include education to the healthcare community and improving hospital systems. The establishment of STEMI systems of care proved to be feasible and effective in Egypt [72]. Females presenting with STEMI should be managed with great vigilance and GDMT should be applied aggressively. Public awareness should target females with chest pain to promote early presentation.

## Conclusions

Critical gender disparities in STEMI care in Egyptian patients, with females presenting at older ages, with a higher burden of comorbidities, and experiencing longer intervals between first medical contact and symptom manifestation. A greater proportion of females were not offered reperfusion treatment, and they exhibited greater in-hospital mortality rates. Hence, efforts to improve early recognition, guideline adherence, and equitable treatment are essential to mitigate these differences.

## Data Availability

No datasets were generated or analyzed during the current study.

## Abbreviations

CVD: Cardiovascular disease
ACS: Acute coronary syndrome
MENA: Middle East and North Africa
STEMI: ST-segment elevation myocardial infarction
NSTEMI: Non-ST-segment elevation myocardial infarction
Gulf RACE: Gulf Registry of Acute Coronary Events
ACCA: Acute cardiovascular care association
EAPCI: European association of percutaneous cardiovascular interventions
EORP: EURObservational research programme
PCI: Percutaneous coronary intervention
LBBB: Left bundle branch block
ECG: Electrocardiogram
FMC: First medical contact
MI: Myocardial infarction
CCU: Coronary care unit
LVEF: Left ventricular ejection fraction
BMS: Bare-metal stent
DES: Drug-eluting stent
AF: Atrial fibrillation
TIA: Transient ischemic attack
MRA: Mineralocorticoid receptor antagonists
PPI: Proton pump inhibitors
DAPT: Dual antiplatelet therapy
CKD: Chronic kidney disease
CAD: Coronary artery disease
MACE: Major adverse cardiovascular events
AMI: Acute myocardial infarction
MINOCA: Myocardial infarction with non-obstructive coronary arteries
SCAD: Spontaneous coronary artery dissection
pPCI: Primary percutaneous coronary intervention
